# In memoriam: Professor Ovidiu Alexandru Bajenaru (1957-2020)

**DOI:** 10.25122/jml-2020-1007

**Published:** 2020

**Authors:** Dafin Muresanu

It is with great grief that we announce the loss of our friend and mentor, Professor Dr. Ovidiu Alexandru Băjenaru. It would be difficult to write of our beloved colleague in these pages without betraying emotion. A brilliant neurologist, researcher, teacher, husband, father, he leaves behind a profound legacy of perhaps the most influential contemporary figure in Romanian neurology.

We remember and cherish some of his most significant achievements over his long and fruitful career. Professor Băjenaru is the founding President of the Romanian Society of Neurology (RSN), an organization that he has formally led for 12 years. In this position, he has contributed decisively to the advancement of science and education in our field. He spearheaded the development of a vision for clinical neurology in our country, based on the conceptual changes determined by colossal advances in neuroscience and modern medical technologies, and integrated using traditional clinical training.

Professor Ovidiu Băjenaru represented Romania at top-notch medical forums. He was Romania’s official representative at the World Federation of Neurology (since 2001), the European Stroke Organization, and the European Union of Medical Specialists (Board of Neurology permanent representative of Romania since 2008). Dr. Băjenaru was also a corresponding member of the Romanian Academy (since 2016), as well as a member of the Romanian Group of Brain Research (since 2016), member in the Bureau of Medical Services (since 2018), member of the Academy of Medical Sciences (since 2013).

His primary areas of interest were neurodegenerative diseases (particularly Parkinson’s disease and Alzheimer’s disease), muscular dystonia and other motor behavior abnormalities, cerebrovascular diseases, multiple sclerosis, and sleep pathology in neurological disorders and medical conditions – all fields in which he became a leading authority. His scientific activity includes over 500 presentations at national and international events and over 50 international multicentric clinical trials as a principal investigator.

Professor Băjenaru coordinated the interdisciplinary medical group for the treatment by deep brain stimulation for Parkinson’s disease (neurology, neurosurgery, medical imaging) from the Bucharest Emergency University Hospital (since 2006), the first Romanian medical group for the interventional treatment of epilepsy (since 2009), and the national health programs for neurological disorders of the National Health Insurance House and the Romanian Ministry of Health (since 2014). Between 2008-2014, he was the President of the Neurology and Pediatric Neurology Commission at the Romanian Ministry of Health.

Among his original scientific contributions, as part his doctoral thesis defended in 1993, he introduced the concept of incipient cognitive impairment for the first time in Romania, referring to patients with cerebrovascular and coronary diseases who develop neurocognitive disorders as a condition preceding the onset of dementia. He has also promoted the implementation of systematic neurocognitive evaluation for patients with neurological and vascular diseases, as well as their treatment in the current pursuit/activity of the neurologists in Romania. Dr. Băjenaru conducted the first brain connectomics study in the context of a neurological condition (Parkinson’s Disease) in Romania. Under the coordination of Professor Băjenaru, a modern school of clinicians and researchers in neurosciences was born, forming several professors and heads of clinics and departments in Bucharest. 

Throughout his career, he has received numerous awards that certify the effort made and the unquestionable dedication which he had in helping those in need. Among the awards and distinctions received we mention the Romanian Patriarchate Award “100 years of Modern Medicine in the capital of united Romania” (2018), The Excellence Award for the entire activity in the field of Neurology in Romania of the Academica Foundation (2016), the Excellence Award for the whole of the activity of the Foundation Prof. Dr. Marin Voiculescu “(2015), Excellence Award of the International Brain Foundation and the Romanian Academy of Medical Sciences - for his activity in the field of neurology in Romania, (2014); Doctor Honoris Causa of the “Ovidius” University of Constanța, Romania (2006); Award of Excellence in Neurology in Romania - for scientific and educational activity at national level, 2005, 2006 and 2011; Romanian Society of Internal Medicine Award for the most active scientific collaborator in a related specialty, 2008, 2012; Excellency prize of the International Society for the Study of Neuroprotection and Neuroplasticity, 2011; Excellence Award for medical activity awarded by the Romanian Ministry of Health in 2015.

He was an influencing neurologist, a fantastic colleague, and a dear friend. It is a great loss for all of us that he has passed away at such a young age. Although he is no longer with us, his powerful impact on our field will last for a very long time. Our deepest sympathy goes to his family in this time of grief.

He will be greatly missed. May his soul rest in peace.

